# Effects of Anti-Inflammatory Treatment and Surgical Intervention on Endothelial Glycocalyx, Peripheral and Coronary Microcirculatory Function and Myocardial Deformation in Inflammatory Bowel Disease Patients: A Two-Arms Two-Stage Clinical Trial

**DOI:** 10.3390/diagnostics11060993

**Published:** 2021-05-30

**Authors:** Charilaos Triantafyllou, Maria Nikolaou, Ignatios Ikonomidis, Giorgos Bamias, Dimitrios Kouretas, Ioanna Andreadou, Maria Tsoumani, John Thymis, Ioannis Papaconstantinou

**Affiliations:** 1 2nd Academic Department of Cardiology, Attikon Hospital, Medical School, National and Kapodistrian University of Athens, 12462 Athens, Greece; ignoik@gmail.com (I.I.); johnythg@gmail.com (J.T.); 2 Department of Cardiology, Amalia Fleming General Hospital of Athens, 15127 Athens, Greece; marianikolaou1974@yahoo.com; 3 GI-Unit, 3rd Academic Department of Internal Medicine, Sotiria Hospital, Medical School, National and Kapodistrian University of Athens, 11527 Athens, Greece; gbamias@gmail.com; 4 Department of Biochemistry and Biotechnology, University of Thessaly, 41500 Larissa, Greece; dkouret@uth.gr; 5 Laboratory of Pharmacology, Faculty of Pharmacy, National and Kapodistrian University of Athens, 15741 Athens, Greece; jandread@pharm.uoa.gr (I.A.); marietsoumani@gmail.com (M.T.); 6 2nd Academic Department of Surgery, Aretaieion Hospital, Medical School, National and Kapodistrian University of Athens, 11528 Athens, Greece; johnpapacon@hotmail.com

**Keywords:** inflammatory bowel disease, Crohn’s disease, ulcerative colitis, inflammation, endothelial dysfunction, atherosclerosis, endothelial glycocalyx, arterial stiffness, oxidative stress

## Abstract

Sixty inflammatory bowel disease (IBD) patients (45 Crohn disease and 15 ulcerative colitis, 40 ± 13 years, 53% male) were examined at baseline and 4 months after intervention (surgical (35 patients) or anti-TNFa treatment (25 patients)). IBD severity, using Mayo score, Harvey–Bradshaw Index (HBI) and biomarkers, was correlated with cardiovascular markers. At baseline, the disease severity, the white blood cells (WBC) values and the reducing power (RP) were significantly correlated with the aortic pulse wave velocity (PWV) (*r* = 0.4, *r* = 0.44 and *r* = 0.48, *p* < 0.05) and the lateral mitral E’ velocity (*r* = 0.35, *p* < 0.05 and *r* = 0.3, *p* < 0.05). Four months after intervention, there was a reduction of WBC (1962.8/mm^3^ ± 0.425/mm^3^, *p* < 0.001), C-reactive protein (CRP) (8.1 mg/L ± 1.7 mg/L, *p* < 0.001), malondialdehyde (MDA) (0.81 nmol/mg ± 0.37, *p* < 0.05) and glycocalyx perfused boundary region (PBR 5-25) (0.24 μm ± 0.05 μm, *p* < 0.01). Moreover, the brachial flow mediated dilatation (FMD), the coronary flow reserve (CFR) and the left ventricle global longitudinal strain (LV GLS) were significantly improved for both groups (4.5% ± 0.9%, 0.55 ± 0.08, 1.4% ± 0.35%, *p* < 0.01), while a more significant improvement of PWV/GLS was noticed in the anti-TNFa group. IBD severity is associated with vascular endothelial, cardiac diastolic, and coronary microcirculatory dysfunction. The systemic inflammatory inhibition and the local surgical intervention lead to significant improvement in endothelial function, coronary microcirculation and myocardial deformation.

## 1. Introduction

Inflammatory bowel diseases (IBD), which are predominantly represented by Crohn’s disease (CD) and ulcerative colitis (UC), constitute a group of chronic and recurrent diseases that involve a deregulation of mucosal immunity and impaired gastrointestinal physiology [1]. Both CD and UC are caused by a combination of genetic, immunologic and environmental factors, which trigger uncontrolled immune responses within the intestine. These uncontrolled responses are characterized by flares and remissions [2].

Due to this inflammatory process, IBD causes functional and structural changes that affect not only the intestinal physiology but also the vascular endothelium, the coronary microcirculation and the left ventricle (LV) systolic and diastolic function. Furthermore, IBD has been associated with an increased risk for stroke, myocardial infarction (MI) and cardiovascular (CV) death, especially during periods of flares (active disease) [3,4,5,6,7].

Over the last years, there has been increasing evidence corroborating a link between IBD and CV dysfunction. More precisely, the IBD population presents an impaired endothelial function and vascular stiffness when assessing markers such as the flow-mediated dilatation (FMD) of brachial artery, the carotid intima-media thickness (cIMT), the aortic pulse wave velocity (PWV) and the augmentation index (AI) [8,9,10,11,12,13,14]. These patients also seem to display a significant relation between their aortic stiffness and the LV systolic and diastolic dysfunction, which predicts an early CV risk [3]. CD is associated with impaired LV global longitudinal strain (GLS) and the disease activity is inversely correlated (negatively correlated) with this cardiac dysfunction [6]. Additionally, active IBD, when compared with the remission period and the healthy group, leads to significant coronary microcirculatory dysfunction [4].

On the other hand, there is limited data regarding the effect of IBD treatment on arterial function and structure. In certain trials, the TNFa (tumour necrosis factor alpha) antibody therapy seems to improve the endothelial dysfunction and vascular stiffness and to downregulate the mucosal angiogenesis in this population [15,16,17,18].

Therefore, this study is designed to examine and identify not only the vascular but cardiac impact as well, of local (surgical) and systemic anti-inflammatory treatment in these patients and to explore the possible differential effects of this intervention between the two approaches.

## 2. Materials and Methods

### 2.1. Study Design and Protocol

A total of sixty IBD patients (45 CD and 15 UC, 40±13 years, 53% male) were examined:

1. at baseline and
2. 4 months after intervention, whereby intervention is herein specifically defined as surgical intervention (35 patients, designated as group A) or pharmaceutical treatment (systemic inflammatory inhibitor (anti-TNFa) for 25 patients, designated as group B).

IBD clinical severity was quantified using the best-known disease activity index scores, Mayo score and Harvey-Bradshaw Index (HBI), for UC and CD, respectively. Mayo is scored on a scale from 0 to 12 which includes the rectal bleeding, the stool frequency, the physician’s global assessment and the endoscopic evaluation (remission 0–1, mild 2–4, moderate 5–6 and severe > 7). HBI score includes the haematocrit level, the body weight and the antidiarrheal medication use (remission <5, mild 5–7, moderate 8–16 and severe > 16) [19,20,21,22]. 

The trial’s inclusion criteria were the following:

- Endoscopic and histologic disease confirmation at least 6 months before admission.
- Uncontrolled inflammatory status, clinically (elevated Mayo—HBI scores) and biochemically (white blood cells (WBC)–c-reactive protein (CRP) values), with frequent recurrences in their classic treatment (salicylates, antibiotics, corticosteroids) or immunomodulatory treatment (methotrexate, azathioprine). Subjects in a stable or improving clinical state were excluded.
- Patients in both groups who had not received, for at least 6 months before admission, any anti-TNFa (tumour necrosis factor alpha inhibitor) or anti-IL (anti-interleukin) agent, with clinical worsening. For this reason, they needed to undergo a new systemic anti-inflammatory medical treatment (group B) in order to eliminate the disease burden or a local surgical approach (group A) because of major intestinal complications such as bowel obstruction, abscesses or fistulas.
- No history of established or first diagnosed—during the baseline visit—cardiovascular disease (CVD) or CV risk factors (diabetes mellitus, dyslipidaemia, arterial hypertension, smoking, family history).

The systemic anti-inflammatory agents that our patients in group B received in this study was a TNFa inhibitor (adalimumab or infliximab), as there is increasing data regarding the efficacy and the safe profile of these agents regarding their use in the IBD population [23,24,25,26].

More specifically, adalimumab is a fully human IgG1 (Immunoglobulin G1) monoclonal antibody that specifically neutralizes the TNFa bioactivity and inducts the apoptosis of TNF-expressing mononuclear cells to TNFa [27]. Infliximab is a chimeric monoclonal IgG1 antibody composed of human constant (75%) and murine variable (25%), which blocks the TNF-α [28,29].

### 2.2. Measurements

#### 2.2.1. Vascular Endothelium Assessment

For the non-invasive evaluation of the vascular endothelial function, we measured the following:

(a) The carotid-femoral pulse wave velocity (cfPWV—Complior SP ALAM), the peripheral brachial PWV, the central systolic blood pressure (cSBP) and augmentation index (AI). PWV is a non-invasive technique that has been accepted as the gold standard procedure to evaluate arterial stiffness and is defined as the velocity at which the pressure waves propagate along the arterial tree. The normal values of PWV are <10 m/s and its increase indicates a higher arterial stiffness in early stages with a significant correlation with CV events [9,10,12,17,30,31,32,33,34,35]. AI is defined as 100 × (P2 − P1)/PP, where P2 is the late backward systolic wave, P1 is the early forward systolic wave, and PP is the pulse pressure. It represents the pressure that is induced by the return of the reflected waves at the aorta [9,10,12,17,30,31,32,33,34,35].

(b) The flow mediated dilatation (FMD) of the brachial artery (Vivid E95 GE Medical Systems, Horten, Norway). Using a linear array transducer (10 MHz), we measured the right brachial artery diameter at end diastole, at baseline and after a shear stress application. We inflated (200–250 mHg) a cuff fitted distally to the brachial artery and after cuff deflation, the hyperaemic arterial blood flow velocity was recorded within the first 90 s, to define its maximal diameter. FMD is calculated by the percentage change of the arterial diameter after hyperaemia from the baseline diameter and is the most popular non-invasive procedure to assess vascular reactivity due to activation of the endothelial nitric oxide (NO) synthase (eNOS) via shear stress [1,2,31,32,33,36,37,38]. 

(c) The perfused boundary region (PBR) of the sublingual arterial microvessels (ranging from 5–25 µm), using Sideview Darkfield imaging (Microscan, Glycocheck, Microvascular Health Solutions Inc., Salt Lake City, UT, USA). Endothelial glycocalyx assists in regulating vascular permeability, as well as in preventing the migration of blood cells to the vessel wall and shear stress transmission. Normally, the endothelial cell is covered by a layer of proteoglycans and glycoproteins and promotes the antioxidant, anti-inflammatory and antithrombotic equilibrium. The direct, non-invasive and fast method of evaluating the PBR of the sublingual arterial microvessels has a very good reproducibility and is proposed as a valid method to assess the endothelial glycocalyx thickness by the European Society of Cardiology Working Group on Peripheral Circulation. Early and rapid alteration of glycocalyx functions has been associated with systemic and local inflammatory processes such as diabetes, atherosclerosis, ischaemia, sepsis, arterial hypertension, smoking, renal failure and psoriasis [31,39,40,41,42,43,44,45,46,47,48].

#### 2.2.2. Echocardiography Measurements

Transthoracic echocardiography exams were performed with GE Vivid I and GE E95 machines (GE Vingmed Ultrasound, Horten, Norway). All images were recorded, encoded and blind reviewed at EchoPac workstation v.201 and v.203 (GE Vingmed Ultrasound, Horten, Norway) by two experienced echocardiographers (I.I, C.T).

We thereby measured:

(a) The LV global longitudinal strain (GLS), the global longitudinal strain rate (GLSR), the longitudinal four chambers strain (L4chS), the global circumferential strain (GcircS) and the PWV/GLS as a marker of ventricular-arterial interaction. Ventricular-arterial interaction assessment possesses independent prognostic and diagnostic value and is used to refine risk stratification and monitor therapeutic interventions especially in inflammatory status and oxidative stress [49,50]. For this purpose, we used the 17 LV myocardial segment model, using the apical 4-, 2-, and 3-chamber views, with a normal value for GLS at −22.5 ± 2.7% [51].

(b) The peak LV twisting, peak twisting velocity (pTwVel) and peak untwisting velocity (pUtwVel), using the parasternal short axis views at basal and apical level in speckle tracking mode. We evaluated the time interval between the onset of the QRS interval of the electrocardiogram (ECG) trace and the onset, peak, and end of the mitral E waveform. Through this method we estimated the peak LV twisting velocity (pTw, deg), as well as the untwisting velocity at the time of mitral valve opening (UtwMVO) and of peak mitral E wave (UtwPEF) [38].

(c) The mitral inflow velocity (E), as well as the mitral annulus velocities (S’ and E’) and the right ventricle (RV) free wall systolic movement by tissue Doppler imaging.

(d) The coronary flow reserve (CFR), through measuring—by Doppler echocardiography—the maximal velocity (CFRv) and the velocity-time integral (CFRvti) in the distal left anterior descending (LAD) coronary artery at baseline and during hyperaemic conditions after intravenous adenosine infusion (0.14 mg/kg per minute) for 3 min. CFR was calculated as the average ratio of three cardiac cycles of hyperaemic to resting maximal diastolic velocity and the same for the velocity-time integral (VTI). CFR values higher than 2.5 are characterized as normal, while values lower than 2 refer to critical epicardial coronary stenosis. CFR values between 2 and 2.5 are considered borderline values that are expressed in coronary microcirculatory damage due to endothelial dysfunction, perivascular fibrosis and elevated filling pressure caused by several extracardiac disorders like hypertension, diabetes and systemic inflammatory diseases [52,53,54,55].

#### 2.2.3. Laboratory Assays

Finally, laboratory assays were conducted, for all patients, concerning: the C-reactive protein (CRP) and the white blood cells (WBC) as inflammatory biomarkers, as well as the total antioxidant capacity (TAC), the thiobarbituric acid reactive substances (TBARS), the ABTS (2,2′-Azino-Bis-3-Ethylbenzothiazoline-6-Sulfonic Acid), the reducing power (RP) and the malondialdehyde (MDA) as oxidative stress biomarkers, using a previously published methodology [56,57,58,59,60,61].

The aforementioned clinical, cardiovascular and laboratory evaluation was performed at baseline as well as 4 months after the intervention and was then correlated with the disease severity index scores.

The study protocol was approved by the institute’s ethics committee and written informed consents were obtained by all patients.

### 2.3. Primary and Secondary Endpoints

The primary endpoints were changes in endothelial glycocalyx thickness (PBR5-25), in coronary microcirculation (CFR), in global LV longitudinal (GLS) and circumferential strain (GcisrS) and in ventricular-arterial interaction, four (4) months after intervention (surgical intervention or anti-inflammatory treatment, in group A or group B respectively). 

Secondary endpoints were changes in endothelial function (FMD), in aortic stiffness (PWV and AI), in LV diastolic function (mitral E/E’, peak untwisting velocities), four (4) months after intervention (surgical intervention or anti-inflammatory treatment, in group A or group B respectively).

### 2.4. Statistical Methodology

Statistical analysis was performed with the Statistical Package for Social Sciences 25.0 for Windows (SPSS Inc., Chicago, IL, USA). Continuous variables are presented as mean ± SD (standard deviation) when normally distributed and as median and interquartile range otherwise. Categorical variables are expressed as percentages of the population. Continuous variables were tested by the Kolmogorov–Smirnov test to assess the normality or not of distribution. Categorical data were analysed using the χ2 test. Independent t-test or Wilcoxon signed-rank test were used for group comparisons. ANOVA (general linear model), with adjustment for age, gender, and interactions for repeated measurements, was applied for measurements of the examined markers at baseline and 4 months *after intervention*. A *p*-value of less than 0.05 (*p* < 0.05) was considered statistically significant.

When the sphericity assumption—as assessed by Mauchly’s test—was not met, the Greenhouse–Geisser correction was used. Post hoc comparisons were performed with Bonferroni’s correction. Correlation between continuous variables was performed using Pearson’s (parametric) or Spearman’s (non-parametric) correlation coefficient.

The odds ratio and 95% confidence intervals (CIs) are presented for the covariates included in the univariable or multivariable logistic regression analysis. 

All tests were two-sided, and a significance level of 5% was used.

## 3. Results

### 3.1. Study Population

In this study, we enrolled a total of sixty IBD patients (45 CD and 15 UC) who had a mean age of 40 ± 13 years. Fifty three percent (53%) of them were of male gender. Thirty-five (35) subjects received a local surgical intervention and were categorized in group A. Twenty-five (25) subjects received pharmaceutical treatment and were categorized in group B. At the initial assessment, the patients had a mean WBC value at 8510/mm^3^ ± 578/mm^3^, CRP at 11.4 mg/L ± 1.8 mg/L and MDA levels at 4.17 ± 0.4 nmol/mg. Their clinical characteristics, the disease severity and the cardiovascular function as well as the initial biomarkers values for all enrolled patients are shown in Table 1.

#### 3.1.1. Vascular Markers and Association with the Disease Activity and the Biomarkers

At the initial assessment, the arterial stiffness as evaluated by the cfPWV was at 9.1 m/s ± 0.3 m/s and the glycocalyx thickness (PBR5-25) at 2.26 μm ± 0.46 μm. In addition, the endothelial vasodilatation by the FMD measurement was 6.73% ± 0.45%. 

**Figure 1:**
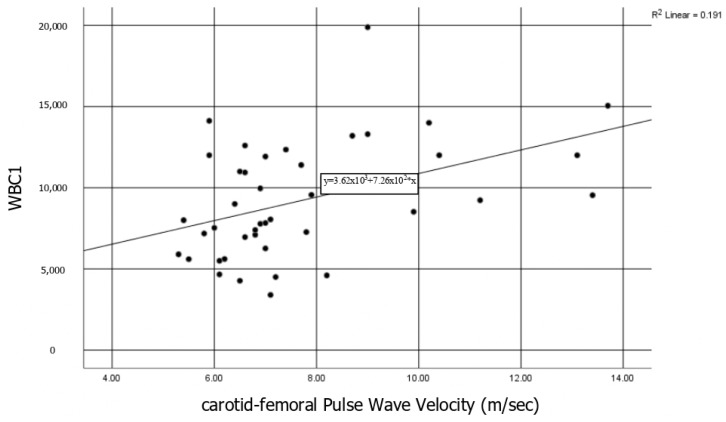
WBC correlation with Pulse Wave Velocity: Baseline correlation of WBC values with the PWV in total population (*r* = 0.4, *p* < 0.05). WBC: white blood cells.

At baseline, the disease severity index score (Mayo score and HBI for UC and CD respectively) and the WBC values were significantly correlated with the peripheral PWV (*r* = 0.4, *p* < 0.05 and *r* = 0.44, *p* < 0.05) (Figure 1). The reducing power (RP) and the ABTS values were correlated with the PWV (*r* = 0.48 and *r* = 0.33 respectively, *p* < 0.05), while the ABTS was associated also with the AI (*r* = 0.34, *p* < 0.05). Finally, the RP was negatively correlated with the FMD (*r* = −0.3, *p* < 0.05).

#### 3.1.2. Cardiac Markers and Association with the Disease Activity and the Biomarkers

The microcirculatory function as assessed by the CFRv was at 2.49 ± 0.06 and by CFRvti at 2.08 ± 0.59. The LV deformation by GLS was −18.9% ± 0.3% and by L4chS −18.8% ± 0.35%, while the ventricular-arterial interaction by calculating the PWV/GLS was −0.49 ± 0.02 m/s%.

At baseline, the WBC was negatively associated with the CFRvti (*r* = −0.26, *p* < 0.05). Additionally, the disease severity index score (Mayo score and HBI for UC and CD respectively) and the WBC values were significantly correlated with the lateral mitral E’ velocity (*r* = 0.35 and *r* = 0.3 respectively, *p* < 0.05). The RP was correlated with the PWV/GLS (*r* = 0.35, *p* < 0.05).

#### 3.1.3. Interrelation between Vascular and Cardiac Markers

**Figure 2:**
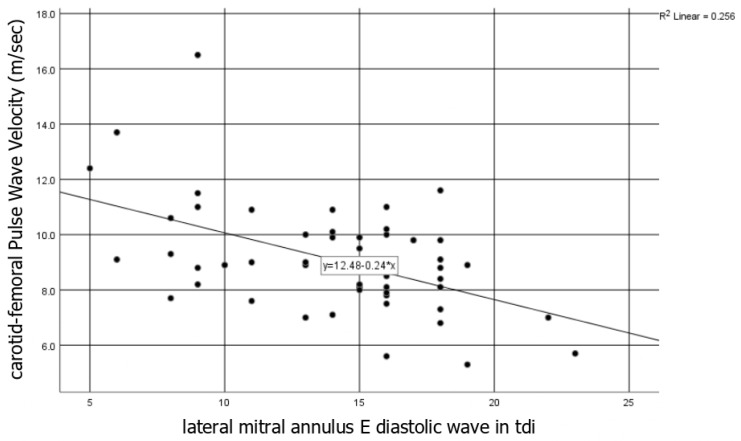
cfPWV correlation with the lateral tissue mitral annulus E diastolic wave: Baseline correlation of central pulse wave velocity (PWV) with the lateral tissue mitral annulus E diastolic wave (*r* = −0.5, *p* < 0.05). tdi: tissue doppler imaging, cfPWV: carotid-femoral pulse wave velocity.

The AI was associated with the lateral and septal mitral E’ velocity (r = −0.6, *p* < 0.05 for both) and with the CFR (*r* = 0.3, *p* < 0.05), while the peripheral and the central aortic PWV was negatively correlated with the lateral mitral E’ velocity (*r* = −0.5, *p* < 0.05) (Figure 2). Moreover, the PWV was negatively correlated with the L4chS (*r* = −0.26, *p* < 0.05).

### 3.2. Effects of Surgical Intervention (Group A) and Pharmaceutical Treatment (Group B)

**Figure 3:**
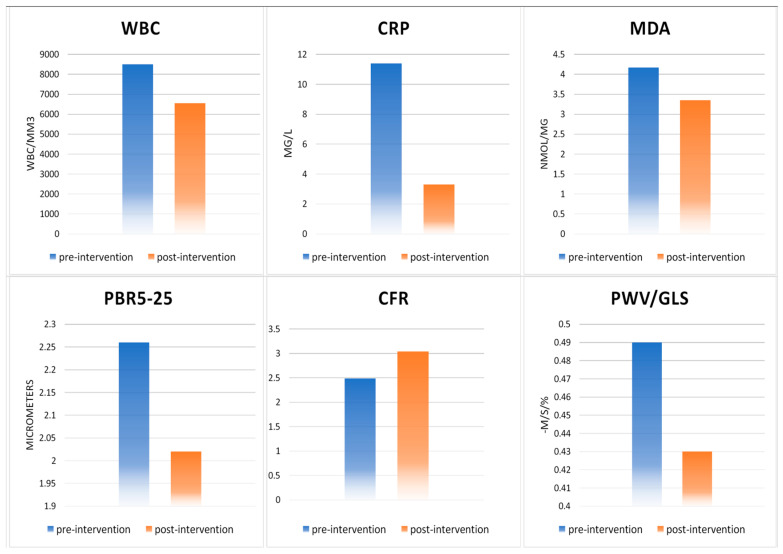
Total population pre-and post-intervention biomarkers, endothelial glycocalyx, coronary micro circulation for WBC, CRP, PBR5-25, CFR changes, *p* < 0.01 for PWV/GLS change and *p* < 0.05 for MDA change. WBC: white blood cells, CRP: C-reactive protein, MAD: malondialdehyde, PBR5-25: perfused boundary region 5–25 µm, CFR: coronary flow reserve, PWV/GLS: ventricular-arterial interaction.

Four months after the initial assessment, there was an overall reduction of WBC values (1962.8/mm^3^ ± 0.425/mm^3^, *p* < 0.001), of CRP (8.1 mg/L ± 1.7 mg/L, *p* < 0.001), of MDA (0.81 ± 0.37 nmol/mg, *p* < 0.05) and of PBR 5–25, 5–9, 10–19 and 20–25 (0.24 μm ± 0.05 μm, 0.06 μm ± 0.02 μm, 0.23 μm ± 0.06 μm and 0.33 μm ± 0.09 μm, respectively, *p* < 0.01 for all changes) (Table 2 and Figure 3).

#### 3.2.1. Vascular Markers and Association with the Disease Activity and the Biomarkers

At the post treatment analysis, we recorded a reduction of PBR 5–25, 5–9, 10–19 and 20–25 (0.24 μm ± 0.05 μm, 0.06 μm ± 0.02 μm, 0.23 μm ± 0.06 μm and 0.33 μm ± 0.09 μm respectively, *p* < 0.01 for all changes), in both groups. Moreover, the FMD was significantly improved (4.5% ± 0.9%, *p* < 0.001).

The changes in WBC values between the pre- and the post-intervention analysis in both groups were significantly correlated with the changes in the PBR 10–19 and PBR 5–9 (*r* = 0.35 and *r* = 0.33, *p* < 0.05 for both). The change in ABTS was negatively correlated with the cSBP changes (*r* = −0.41, *p* < 0.050).

Furthermore, the change in the CRP value was significantly correlated with the change in the FMD (*r* = 0.35, *p* < 0.05).

#### 3.2.2. Cardiac Markers and Association with the Disease Activity and the Biomarkers

**Figure 4:**
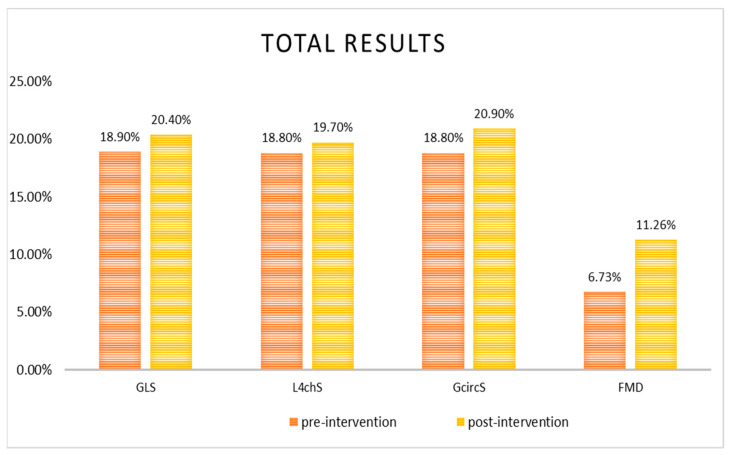
Total population pre-and post-intervention cardiac deformation and vascular dilatation results: *p* < 0.001 for GLS changes and *p* < 0.01 for L4chS, GcircS and FMD changes. GLS: global longitudinal strain, L4chS: longitudinal four chambers strain, GcircS: global circumferential strain, FMD: flow-mediated vasodilatation.

Four months after the initial assessment, the RV free wall systolic movement and the CFR were significantly improved (6.5 mm ± 2.6 mm and 0.55 ± 0.08, *p* < 0.001). The LV GLS and L4chS, as well as the GcircS and the PWV/GLS were significantly improved as well (1.4% ± 0.35%, 0.86% ± 0.23%, 2.2% ± 0.37% and 0.05 ± 0.01, *p* < 0.01 for all changes). Finally, an overall improvement of UtwPEF (39.5^°^/s ± 14.5^°^/s, *p* < 0.05) was noticed (Figure 4).

An interaction was recorded between the type of intervention (medical vs. surgical) and the change of PWV/GLS (*p* = 0.04). More specifically, the change of ventricular-arterial interaction in the pharmaceutically treated patients (Group B) at the four months’ assessment, was greater compared to the surgically treated subjects (0.082 ± 0.02, *p* = 0.03 vs 0.033 ± 0.02, *p* = 0.04).

The changes in WBC values between the pre- and the post-intervention analysis in both groups were significantly correlated with the changes in the lateral mitral E’ (*r* = −0.36, *p* < 0.05), with the changes in the CFR (*r* = 0.36, *p* < 0.05) as well as with the changes in the GcircS (*r* = 0.36, *p* < 0.05). Moreover, the changes in CFR were negatively correlated with the ABTS changes (*r* = −0.69, *p* < 0.05), while the change in the CRP value was significantly correlated with the change in the lateral mitral E’ (*r* = −0.36, *p* < 0.05).

## 4. Discussion

### 4.1. Baseline Characteristics

#### 4.1.1. Baseline Vascular Markers Analysis

The findings of the present study corroborate the available preexisting data that inflammatory bowel disease severity is associated with a significant vascular impairment [8,9,10,11,12,14,17,30,34,35,62,63,64,65,66,67,68,69,70,71,72]. The enrolled IBD subjects presented an advanced systemic inflammatory status in both groups. This inflammatory process affected the endothelial structure and function, as evaluated by the impaired glycocalyx thickness and vasodilatory responsiveness by flow mediated dilatation assessment, as well as the arterial stiffness, by measuring the carotid-femoral PWV. The disease severity and the WBC values were also significantly correlated with the peripheral PWV, indicating the inflammation consequences on arterial stiffness.

An impaired PWV is observed in conditions which are traditionally associated with vascular endothelial dysfunction such as coronary artery disease, peripheral vascular disease, diabetes, dyslipidemia and smoking, as well as with systematic inflammatory diseases such as rheumatoid arthritis, ankylosing spondylitis and IBD. Patients whose baseline PWV was affected were reclassified into a higher risk category of cardiovascular morbidity. In addition, particularly in the case of systematic inflammatory diseases, studies of the past 15 years have demonstrated a statistically significant improvement of the PWV in this category of patients after systemic inflammatory inhibition [9,10,12,17,30,31,32,33,34,35,73,74,75]. 

#### 4.1.2. Baseline Cardiac Markers Analysis 

Moreover, this study indicates the impact of inflammation on cardiac function. The IBD population in this study presents an impaired LV diastolic function, as well as a decrease of LV deformation index, which is positively correlated with the severity of the disease and the inflammatory status, possibly through the affected coronary microcirculatory function. 

GLS constitutes a measure of systolic function and is used as a more sensitive marker than LVEF (left ventricular ejection fraction) for the investigation of the subclinical LV dysfunction. Thus, it has been observed that it is affected not only in coronary artery diseases and in heart diseases, but also in untreated hypertensive or diabetic patients [44,76]. In addition, GLS possesses a prognostic character for heart failure events, the adverse LV remodeling and cardiovascular death. It appears that in the case of anti-remodeling treatments there is an improvement of the GLS, before the appearance of this impact on the impaired LVEF [77,78]. Likewise, studies have demonstrated the correlation of anti-inflammatory treatment (anti-inflammatory inhibition) with an improvement of the LV deformation index, for example in patients with rheumatoid arthritis or psoriasis after IL-1 or IL-12 inhibition, respectively [38,79,80,81].

Additionally, the disease severity and the WBC values were significantly correlated with the lateral mitral E’, highlighting the effects of chronic inflammatory process in diastolic dysfunction. These results confirm previous study findings that the IBD population presents a diastolic dysfunction, an impaired coronary microcirculation and myocardial deformation [3,4,5,6]. 

Furthermore, the arterial stiffness index was negatively correlated with the lateral mitral E’. The Doppler assessment of distal LAD, before and after intravenous adenosine infusion, showed a significant impairment of coronary microcirculatory function. In addition, we observed a significant reduction of global LV longitudinal and circumferential strain in trial subjects at the initial evaluation of both groups.

#### 4.1.3. Analysis of Overall Baseline Results

In attempting to provide a possible explanation for the aforementioned baseline results of our study we would like to highlight certain available data of previous studies on the subject: from a pathophysiological point of view, systemic inflammation triggers a number of structural and functional differences in the vascular endothelium, disrupting the balance between vasodilators and vasoconstrictors, inflammatory cytokines and adhesion molecules. This process promotes platelet aggregation, thrombus formation and peripheral ischemia, as well as inflammatory angiogenesis and hypoxia. Moreover, the reduction of available NO leads to a disruption of normal vasodilation and relaxation [1,2,5,37]. Through these mechanisms, a number of chronic inflammatory diseases lead to an increased vascular stiffness and endothelial dysfunction, and finally to early atherosclerotic disease [31,32,33,43,44,79,82,83,84,85]. 

In recent years, a variety of clinical and experimental studies have focused on the residual cardiovascular risk and the contribution of inflammation to the presence and progression of cardiovascular disease. These trials reveal a significantly influenced endothelial vasodilatory function and arterial stiffness [14,35,70]. At the same time, studies also point to a strong association between chronic inflammatory process and impaired coronary microcirculation, left ventricular diastolic function, longitudinal myocardial strain and strain rate (GLSR) at levels comparable to coronary patients [3,4,6,86].

In conclusion, the overall analysis of the results at the initial assessment demonstrates the impact of the chronic systemic inflammation in the IBD population on the cardiovascular dysfunction for both groups. 

### 4.2. Post-Intervention Analysis

#### 4.2.1. Post-Intervention Vascular Markers Analysis

Following processing of the results after the anti-inflammatory treatment, the analysis of inflammatory and oxidative stress biomarkers revealed a significant overall reduction of inflammatory burden, which leads to significant improvement of endothelial dysfunction in both groups. More specifically, 4 months after intervention, the patients presented a significant reduction of glycocalyx thickness, as measured by PBR assessment and a significant improvement of the endothelial vasodilatory response assessing the flow mediated vasodilatation of brachial artery.

#### 4.2.2. Post-Intervention Cardiac Markers Analysis

Furthermore, we observed in both groups an overall improvement of LV deformation index, probably through the impact of coronary circulatory improvement after the anti-inflammatory treatment. The subjects presented a significant increase of LV global and four chambers longitudinal and circumferential strain. Additionally, the changes in the WBC values were significantly correlated with the improvement in the coronary microcirculation and the diastolic function. Finally, the present study demonstrated an overall improvement of ventricular-arterial interaction, which was identified by measuring the ratio of central aortic PWV to LV GLS (a non-invasive measurement of the ratio of arterial (Ea) to ventricular end-systolic elastance (Ees)). This result seems particularly important in approaching and refining cardiovascular risk stratification in this population and monitoring therapeutic interventions, as an independent diagnostic and prognostic marker.

#### 4.2.3. Group Analysis 

As regards the group comparison, an important finding after the group analysis was that the systemic inflammatory inhibition leads to a significant reduction of inflammatory burden in both groups. Moreover, the present study concluded that this inflammatory post-intervention reduction was significantly correlated with the improvement of endothelial function especially via the endothelial glycocalyx assessment, as well as with the improvement of coronary microcirculation and cardiac deformation index, without statistical differences between the two groups. 

However, the change in ventricular-arterial interaction was greater after the pharmaceutical therapy (group B) when compared to surgery (group A), as regards the changes in the ventricular and particularly in the arterial component. This last result could possibly be explained by the interval period between the intervention and the time point when the subjects were examined and the possible differential time effect of the systemic TNFa inhibition and the surgical intervention on the arterial PWV as well as on the LV deformation. The improvement in the 4-month post-intervention analysis, was more significant for the pharmaceutically treated patients and this is a point that could be reassessed at a more remote time point, for example a year after intervention.

In this field, regarding the link between endothelial dysfunction and IBD treatment, there is up to present extremely limited data indicating the improvement after a TNFa inhibition [15,16,17,18]. Nonetheless, the present clinical trial explores not only the link between the improvement of the endothelial function after systemic inflammatory inhibition but also the impact on the cardiac function. Additionally, it is designed to compare the results between the two studied groups in the post-intervention analysis.

Furthermore, the statistical analysis and comparison between the two groups concluded that there was a significant improvement with regard to endothelial structure and function, central arterial stiffness, coronary microcirculation and cardiac deformation and diastolic index. 

The aforementioned group analysis results could to some extent be explained through the hypothesis that the surgical intervention (group A) may lead to a kind of systemic reduction of excess inflammatory burden at a level which is comparable to the one recorded for the patients receiving systematic anti-inflammatory pharmaceutical treatment (group B).

#### 4.2.4. Analysis of Post-Intervention Results

At this point, it is useful to make reference to the available data supporting that the inflammatory pathways impact the pathogenesis of several chronic diseases. Inflammatory stimuli activate intracellular signals that then activate the production of inflammatory mediators. Inflammation progresses by the action of microbial products and pro-inflammatory cytokines such as interleukin-1β (IL-1β), interleukin-6 (IL-6), tumor necrosis factor-α (TNF-α), gamma-interferon (IFNγ), interleukin-12 (IL-12) and interleukin-18 (IL-18) [87,88]. Inflammasome is a cytoplasmic multi-protein complex that senses exogenous and endogenous danger signals and cleaves pro-inflammatory cytokines into mature cytokines such as IL-1β and IL-18 [89,90,91].

The inhibition of this cascade in any phase may lead to minimizing or totally stopping the inflammatory process [92,93,94]. Newer studies have recently highlighted therapeutic interventions to combat the inflammatory process and vascular dysfunction, with the main representatives being the TNFα inhibitors, the anti-IL1, anti-IL6 and anti-IL12 / 23. They demonstrate the beneficial effect of biological agents (anti-IL1 (Anakinra), anti-IL1b (Canacinumab), anti-IL6 (tocilizumab), anti-IL12 / 23 (ustekinumab)) on the improvement of the endothelium [16,17,18,38,80,95,96,97,98].

This systemic inhibition in these inflammatory pathways could be an explanation for the significant cardiovascular improvement recorded in group B of our study. 

On the other hand, the same significant results can be observed in the surgically treated patients 4 months after the surgical intervention. A higher value in the LV deformation index and the central arterial PWV for the pharmaceutically treated patients remains to be investigated at a more remote time point after the intervention. 

These results could conclude that the resection of an intestinal inflammatory part can lead to a systemic inflammatory inhibition comparable to the inhibition caused by anti-TNFa treatment in the IBD population in all cardiovascular markers that were examined and analyzed in this study.

### 4.3. Additional Remarks for Potential Consideration in Future Research

It is considered pertinent at this point to make reference to probiotics, which are used in several patient groups with intestinal gut diseases as dietary supplements. There appears to be a growing literature highlighting the cardiovascular benefits of the use of probiotics and increasing data associating probiotics with a significant reduction of certain CV risk factors such as low-density lipoprotein cholesterol (LDL-C), total cholesterol, blood pressure, triglycerides, body mass index (BMI) and waist circumference [99,100,101,102,103,104,105]. Research also indicates that probiotics could potentially have a beneficial effect on IBD [106,107,108], yet more clinical trials are needed in this area [109,110,111]. Recent studies have also demonstrated the benefits of pre- and probiotics in the inflammation cascade. The role of pre- and probiotics in decelerating the persistent low-grade chronic inflammation could form the basis and scope of further clinical trials and possibly contribute to their future use as a new supplementary therapeutic means, in addition to the basic anti-inflammatory treatment, possibly with beneficial outcomes for this specific category of patients [112].

Furthermore, we have observed in both groups a statistically significant correlation between the improvement in the disease severity indexes (HBI and Mayo score) and the improvement in the cardiovascular markers, particularly with regard to the improvement of PWV, CFR, GLS and the ventricular—arterial interaction by calculating the PWV-GLS four months after the intervention. These disease severity indexes include—besides clinical data – also a scoring of the patient’s overall health status, including his or her own perception of well-being. While the psychological impact and particularly patients’ perception of well-being appears to be a less investigated area, principally because of its subjective component, recent research has been highlighting the importance of investigating this concept as well, through the use of quantifiable methodological parameters, with the aim to assist in the development of therapeutic interventions that are more comprehensively adapted to patients’ needs [113].

### 4.4. Summary

The results of this clinical trial, via studying IBD population, could have an impact on the prognosis and management of more inflammatory diseases, especially if further research activities elaborate and expand on these findings. We hope that this clinical trial will be the stimulus for more effort in this particularly important field about the interaction of systemic inflammation and cardiovascular disease, for the purpose of improving the pharmaceutical approach and the surgical techniques to mitigate and stop this burden, in order to improve the overall cardiovascular system [97,98,114,115]. 

Moreover, the combination of a surgical targeted approach as well as systemic pharmaceutical inflammatory inhibition seems to produce the optimal results in this population.

It is important to note that an increasing number of studies strongly correlate the presence of a chronic systemic inflammatory process with endothelial dysfunction, early atherosclerosis, and accelerated cardiovascular morbidity, through pathophysiological pathways beyond the classic knowledge about cardiovascular risk factors. Therefore, we are convinced that therapeutic targeting in this direction can make a significant contribution to more efficiently and comprehensively treating cardiovascular events in this context. A larger number of well-designed clinical trials are needed to establish safer conclusions and more consistent treatment options.

### 4.5. Limitations

A limitation of this study was the modest number of enrolled subjects especially for the group of pharmaceutically treated patients. This has limited the possibility of assessing the effectiveness of each agent separately and of specifying the correlation between them. An increase in the number of pharmaceutically treated patients could further enhance the results of the present study. Furthermore, the assessment of the study population 4 months after intervention does not explore the long-term cardiovascular evolution in the two study groups and further comparison possibilities between them, which could be addressed in future research. A long-term follow-up, to rule out clinical effects, could provide interesting findings and results, which could potentially also further support the data of our four-month follow-up. In the light of the above, prospective large-scale studies are needed to investigate this issue further.

## 5. Conclusions

Inflammatory bowel disease severity is associated with vascular endothelial, cardiac diastolic and coronary microcirculatory dysfunction. The systemic inflammatory inhibition leads to significant improvement in myocardial deformation, endothelial and coronary microcirculatory function possibly through a systemic reduction of excess inflammatory burden. The local surgical intervention can also lead to an inflammatory inhibition comparable to the inhibition caused by anti-TNFa treatment in the IBD population.

## Figures and Tables

**Table 1:** Population baseline characteristics.

Characteristics/Markers	Total Population	Surgical Group (A)	Pharmaceutical Group (B)
Enrolled patients	60	35	25
Male	32 (53%)	19 (54%)	13 (52%)
Female	28 (47%)	16 (46%)	12 (48%)
Crohn Disease	46 (77%)	30 (86%)	16 (64%)
Ulcerative Colitis	14 (23%)	5 (14%)	9 (36%)
Age, y	40 ± 13	40.5 ± 13.7	39.8 ± 12.5
WBC, /mm^3^	8510 ± 578	8801 ± 640	7990 ± 1156
CRP, mg/L	11.4 ± 1.8	11.9 ± 2.5	10.6 ± 2.6
MDA, nmol/mg	4.17 ± 0.4	4.04 ± 0.53	4.36 ± 0.63
TBARS, μmol/L	4.13 ± 0.52	4.32 ± 0.92	3.93 ± 0.54
ABTS, mmol/L	24.9 ± 0.96	25 ± 1.42	24.8 ± 1.35
RP, μmol/mL	0.97 ± 0.17	0.96 ± 0.02	0.98 ± 0.03
PWV peripheral, m/s	7.5 ± 0.48	7.44 ± 0.75	7.58 ± 0.5
PWV central, m/s	9.1 ± 0.3	8.7 ± 0.28	9.86 ± 0.62
PBR5-25, μm	2.26 ± 0.46	2.23 ± 0.06	2.31 ± 0.07
PBR5-9, μm	1.22 ± 0.15	1.22 ± 0.02	1.21 ± 0.02
PBR10-19, μm	2.4 ± 0.05	2.38 ± 0.07	2.44 ± 0.08
PBR20-25, μm	2.87 ± 0.08	2.83 ± 0.1	2.94 ± 0.1
CFRv	2.49 ± 0.06	2.45 ± 0.07	2.55 ± 0.09
CFRvti	2.08 ± 0.06	1.97 ± 0.06	2.27 ± 0.1
FMD, %	6.73 ± 0.45	6.73 ± 0.55	6.74 ± 0.81
GLS, %	−18.9 ± 0.3	−19.5 ± 0.39	−18 ± 0.44
PWV/GLS, m/s%	−0.49 ± 0.02	−0.45 ± 0.02	−0.55 ± 0.03
L4chS, %	−18.8 ± 0.35	−19.1 ± 0.4	−18.2 ± 0.64
GcircS, %	−18.8 ± 0.56	−18.7 ± 0.62	−18.9 ± 1.1

WBC: white blood cells; CRP: C-reactive protein; MDA: malondialdehyde; TBARS: thiobarbituric acid reactive substances; ABTS: 2,2′-Azino-Bis-3-Ethylbenzothiazoline-6-Sulfonic Acid; RP: reducing power; PWV: pulse wave velocity; PBR: perfused boundary region; CFRv: coronary flow reserve velocity; CFRvti: coronary flow reserve velocity-time integral;FMD: flow-mediated vasodilatation; GLS: global longitudinal strain; PWV/GLS: ventricular-arterial interaction; L4chS: longitudinal four chambers strain; GcircS: global circumferential strain; y: year.

**Table 2:** Changes in inflammation, oxidative stress, endothelial and cardiovascular markers in the study population during the study period.

Markers	Total Population		Surgical Group (A)		Pharmaceutical Group (B)	
	Pre-Treatment	Post-Treatment	Pre-Treatment	Post-Treatment	Pre-Treatment	Post-Treatment
WBC, /mm^3^	8510 ± 578	6547 ± 314 ^‡^	8801 ± 640	6792 ± 420 ^‡^	7990 ± 1156	6110 ± 445 *
CRP, mg/L	11.4 ± 1.8	3.3 ± 0.8 ^‡^	11.9 ± 2.5	3.8 ± 1.2 ^†^	10.6 ± 2.6	2.3 ± 0.56 *
MDA, nmol/mg	4.17 ± 0.4	3.35 ± 0.26 *	4.04 ± 0.53	3.22 ± 0.3	4.36 ± 0.63	3.57 ± 0.47 *
TBARS, μmol/L	4.13 ± 0.52	4.07 ± 0.48	4.32 ± 0.92	3.98 ± 0.82	3.93 ± 0.54	4.15 ± 0.54
ABTS, mmol/L	24.9 ± 0.96	25.9 ± 1.17	25 ± 1.42	25 ± 1.95	24.8 ± 1.35	26.8 ± 1.26
RP, μmol/mL	0.97 ± 0.17	0.96 ± 0.16	0.96 ± 0.02	0.94 ± 0.01	0.98 ± 0.03	0.99 ± 0.03
PWV peripheral, m/s	7.5 ± 0.48	7.16 ± 0.33	7.44 ± 0.75	7.14 ± 0.5	7.58 ± 0.5	7.17 ± 0.4
PWV central, m/s	9.1 ± 0.3	8.8 ± 0.3	8.7 ± 0.28	8.7 ± 0.37	9.86 ± 0.62	9.1 ± 0.55 *
PBR5-25, μm	2.26 ± 0.46	2.02 ± 0.45 ^‡^	2.23 ± 0.06	2.02 ± 0.05 ^†^	2.31 ± 0.07	2.02 ± 0.08 †
PBR5-9, μm	1.22 ± 0.15	1.15 ± 0.17 *	1.22 ± 0.02	1.16 ± 0.02 *	1.21 ± 0.02	1.14 ± 0.03 *
PBR10-19, μm	2.4 ± 0.05	2.18 ± 0.06 ^†^	2.38 ± 0.07	2.18 ± 0.07 *	2.44 ± 0.08	2.17 ± 0.1 *
PBR20-25, μm	2.87 ± 0.08	2.55 ± 0.07 ^†^	2.83 ± 0.1	2.51 ± 0.07 *	2.94 ± 0.1	2.6 ± 0.1 *
CFRv	2.49 ± 0.06	3.05 ± 0.08 ^‡^	2.45 ± 0.07	3.1 ± 0.1 ^‡^	2.55 ± 0.09	2.96 ± 0.14 †
CFRvti	2.08 ± 0.06	2.47 ± 0.0 6 ^‡^	1.97 ± 0.06	2.43 ± 0.63 ^‡^	2.27 ± 0.1	2.54 ± 0.1 *
FMD, %	6.73 ± 0.45	11.26 ± 0.95 ^‡^	6.73 ± 0.55	12.75 ± 1.27 ^‡^	6.74 ± 0.81	8.8 ± 1.2
GLS, %	−18.9 ± 0.3	−20.4 ± 0.3 ^‡^	−19.5 ± 0.39	−20.7 ± 0.38 *	−18 ± 0.44	−19.8 ± 0.43 †
PWV/GLS, m/s%	−0.49 ± 0.02	−0.43 ± 0.02 ^†^	−0.45 ± 0.02	−0.42 ± 0.02 *	−0.55 ± 0.03	−0.47 ± 0.03 *
L4chs, %	−18.8 ± 0.35	−19.7 ± 0.32 ^†^	−19.1 ± 0.4	−19.8 ± 0.4 *	−18.2 ± 0.64	−19.4 ± 0.55 *
GcircS, %	−18.8 ± 0.56	−20.9 ± 0.68 ^†^	−18.7 ± 0.62	−20.6 ± 0.82 *	−18.9 ± 1.1	−21.6 ± 1.2 *

Data are expressed as median values (first quartile–third quartile) or mean values ± SD. * *p* < 0.05, ^†^ *p* < 0.01 and ^‡^ *p* < 0.001 for time x treatment interaction obtained by repeated-measures ANOVA, for comparisons of 4 months vs baseline. WBC: white blood cells; CRP: C-reactive protein; MDA: malondialdehyde; TBARS: thiobarbituric acid reactive substances; ABTS: 2,2′-Azino-Bis-3-Ethylbenzothiazoline-6-Sulfonic Acid; RP: reducing power; PWV: pulse wave velocity; PBR: perfused boundary region; CFRv: coronary flow reserve velocity; CFRv: coronary flow reserve velocity-time integral; FMD: flow-mediated vasodilatation; GLS: global longitudinal strain; PWV/GLS: ventricular-arterial interaction; L4chS: longitudinal four chambers strain; GcircS: global circumferential strain.

## Data Availability

The datasets analyzed during the current study are available from the corresponding author on request.

## Abbreviations

ABTS	2, 2′-Azino-Bis-3-Ethylbenzothiazoline-6-Sulfonic Acid
AI	augmentation index
anti-IL	anti-interleukin
anti-IL1	anti-interleukin-1
anti-IL12	anti-interleukin-12
anti-IL6	anti-interleukin-6
anti-TNFa	tumour necrosis factor alpha inhibitor
CD	Crohn’s disease
cfPWV	carotid-femoral pulse wave velocity
CFR	coronary flow reserve
CFRv	coronary flow reserve velocity
CFRvti	coronary flow reserve velocity-time integral
cIMT	carotid intima-media thickness
CIs	confidence intervals
CRP	C-reactive protein
cSBP	central systolic blood pressure
CV	cardiovascular
CVD	cardiovascular disease
CRP	c-reactive protein
Ea	arterial elastance
ECG	electrocardiogram
Ees	end-systolic elastance
eNOS	endothelial nitric oxide synthase
FMD	flow-mediated vasodilatation
GcircS	global circumferential strain
GLS	global longitudinal strain
GLSR	global longitudinal strain rate
HBI	Harvey-Bradshaw Index
IBD	inflammatory bowel diseases
IFN-γ	gamma-interferon
IgG	Immunoglobulin G
IL-12	interleukin-12
IL-18	interleukin-18
IL-1β	interleukin-1β
IL-23	interleukin-23
L4chS	longitudinal four chambers strain
LAD	left anterior descending
LV	left ventricle
LVEF	left ventricular ejection fraction
MAdCAM-1	Mucosal vascular addressin cell adhesion molecule 1
MDA	malondialdehyde
MI	myocardial infarction
NO	nitric oxide
P1	early forward systolic wave
P2	late backward systolic wave
PBR	perfused boundary region
PP	pulse pressure
pTw	peak twisting
pTwVel	peak twisting velocity
pUtwVel	peak untwisting velocity
PWV	pulse wave velocity
RP	reducing power
RV	right ventricle
SCBP	systolic central blood pressure
SD	standard deviation
TAC	total antioxidant capacity
tdi	tissue doppler imaging
TBARS	thiobarbituric acid reactive substances
TNFa	tumour necrosis factor alpha
TNF	tumour necrosis factor
UC	ulcerative colitis
UtwMVO	untwisting velocity at the time of mitral valve opening
UtwPEF	untwisting velocity at the time of peak mitral E wave
VTI	velocity-time integral
WBC	white blood cells
